# A commentary on ‘Circulating 25-hydroxyvitamin D concentration can predict bowel resection risk among individuals with inflammatory bowel disease in a longitudinal cohort with 13 years of follow-up’

**DOI:** 10.1097/JS9.0000000000001494

**Published:** 2024-04-19

**Authors:** Biao Liang, Yiheng Yang, Zhenyi Wang

**Affiliations:** Department of Coloproctology, Yueyang Hospital of Integrated Traditional Chinese and Western Medicine, Shanghai University of Traditional Chinese Medicine, Shanghai, People’s Republic of China

*Dear Editor,*

Inflammatory bowel disease (IBD), including ulcerative colitis (UC) and Crohn’s disease (CD), is currently an incurable chronic and disabling bowel disease. The global incidence of IBD is increasing and represents a major challenge for public health and the management of IBD^1^. Bowel resection is a key clinical outcome to improve the long-term prognosis of IBD^2^. Revealing relevant risk factors is essential for the identification of people at risk for bowel resection during the long-term management of IBD. Increasing vitamin D levels significantly lowers C-reactive protein levels in patients with IBD and reduces intestinal inflammation in patients with IBD^3^. Furthermore, decreasing serum 25(OH)D levels generally increases the risk of surgery in patients with UC^4^. The exploration of the association between serum 25(OH)D concentration and the risk of bowel resection in individuals with IBD carries considerable significance.

We would like to express our sincere appreciation to Dan Lintao and his coworkers for their insightful and valuable paper published in the *International Journal of Surgery* ^5^. They have conducted a longitudinal cohort study of 5474 individuals with IBD in the UK Biobank. Their comprehensive study addresses the complex relationship between serum 25(OH)D levels and the risk of bowel resection in patients with IBD, representing a commendable effort to help clinicians predict and screen for surgical events. We make insightful recommendations in the expectation of further improving the quality of this article.

Firstly, regarding the exclusion criteria, although the authors had set strict inclusion and exclusion criteria for participants, we recommend adding the following criteria: ‘1. individuals with previous bowel resections’ and ‘2. individuals with colorectal cancer.’ These considerations are crucial as they may increase the risk of bowel resection, which ultimately affects the assessment of serum 25(OH)D concentration with the risk of bowel resection in patients with IBD.

Secondly, to reduce the possibility of confounding variables influencing results, this study meticulously corrected several significant confounders. In this study, serum 25(OH)D concentration was set as the exposure variable and bowel resection as the outcome variable. The sociodemographic factors in the study include sex, age, ethnic background, education level, BMI, TDI, smoking status, alcohol status, diet, and physical activities. Factors directly associated with the exposure variable include sunlight exposure, blood sampling seasons, vitamin D supplement use, and multivitamin supplement use. Other covariates include inflammatory indicators, baseline comorbidities, and regular IBD-related medication use. Although the authors adjusted for several covariates as rigorously and comprehensively as possible, we recommend increased consideration of other potential confounders. These include income, marital status, fecal calprotectin, serum iron concentration, opioid use in IBD, and psychological comorbidity. This would further increase the reliability and robustness of the study results.

Finally, this study was designed as an observational study with a low level of evidence. Further studies with a higher level of evidence, such as randomized controlled trials, are needed to confirm the potential causal relationship between vitamin D deficiency and adverse surgical outcomes in IBD.

In conclusion, the study by Dan Lintao *et al*. significantly advances our understanding by providing a comprehensive perspective on the association between serum 25(OH)D concentration and the risk of bowel resection in IBD. This study suggests that an elevated serum 25(OH)D level is independently associated with a lower risk of bowel resection in IBD, which presents as an inverse linear dose–response relationship. Vitamin D deficiency may be an effective predictor of the risk of bowel resection in the management of IBD. We are grateful for the opportunity to provide feedback on this study and look forward to the further contributions of Dan Lintao and others to the IBD study and our suggestions for its continued scientific improvement.

## Ethical approval

There are no patients involved in this manuscript, therefore no ethical approval needed.

## Sources of funding

This study was supported by the National Natural Science Foundation of China (Nos 82274531 and 81973859).

## Author contribution

B.L.: wrote and revised the contents of this manuscript and submitted it for publication; Y.Y.: conducted initial revisions of this manuscript; Z.W.: proposed the core concepts in this manuscript, organized the structure, and conducted revisions and reviews of this manuscript.

## Conflicts of interest disclosure

The authors declare no conflicts of interest.

## Research registration unique identifying number (UIN)

There are no patients involved in this manuscript, therefore no clinical research registration.

## Guarantor

The guarantor of this manuscript is Zhenyi Wang. He is familiar with the content and design of the article and can act as a guarantor of the article.

## Data availability statement

No data were generated during the writing of this study.
